# Complex Regional Pain Syndrome (CRPS) and the Value of Early Detection

**DOI:** 10.1007/s11916-023-01124-3

**Published:** 2023-07-06

**Authors:** Michael Alexander Harnik, Pascal Kesselring, Alexander Ott, Richard D. Urman, Markus M. Luedi

**Affiliations:** 1 Department of Anaesthesiology and Pain Medicine, Inselspital, Bern University Hospital, University of Bern, Bern, Switzerland; 2 Department of Anaesthesiology and Pain Medicine, Cantonal Hospital of St. Gallen, St. Gallen, Switzerland; 3 Department of Anaesthesiology, College of Medicine, The Ohio State University, Columbus, OH 43210 USA

**Keywords:** CRPS, Treatment, Diagnosis, Recognition, Prevention, Awareness

## Abstract

**Purpose of Review:**

The goal of this narrative review is to describe the current understanding of the pathology of Complex Regional Pain Syndrome (CRPS), as well as diagnostic standards and therapeutic options. We will then make the case for early recognition and management.

**Recent Findings:**

CRPS remains an enigmatic pain syndrome, comprising several subtypes. Recent recommendations clarify diagnostic ambiguities and emphasize the importance of standardized assessment and therapy.

**Summary:**

Awareness of CRPS should be raised to promote prevention, early detection, and rapid escalation of therapy in refractory cases. Comorbidities and health costs (i.e., the socioeconomic impact) must also be addressed early to prevent negative consequences for patients.

## Background

Among “mysterious” posttraumatic pain phenomena, complex regional pain syndrome (CRPS) is certainly one of the greatest challenges clinicians face in their daily practice. It is considered a rare but severe complication of injuries to the extremities that can progress into a debilitating form of persistent pain. Early diagnosis and treatment are deemed crucial to avoid worse outcomes. In this review, we will outline the pathophysiology and examine recent developments in diagnosis and standard treatment of the condition. Further, we will lay out the importance of early detection and care.

## Epidemiology and Clinical Presentation

The two most cited, population-based studies place the incidence of CRPS between 5.5 and 26.2 per 100.000 person years [1, 2]. The difference between the two figures reflects the nature of a rare diagnosis that depends on the population and study setting in which the data are collected. Women were about four times more likely to be affected (with a female-to-male ratio of 3.4–4 to 1) with a mean age at onset of 46.9 to 52.7 years, where another study places the highest peaks between 45 and 55 years [3]. Trauma is a common trigger with reported incidences of 4.36% for foot or ankle fractures with surgery [4] or even 8.8% for distal radius fractures, reflecting the observation that the upper extremities are more often affected [5]. We can divide the condition into type I (formerly known as Sudeck’s dystrophy, without nerve injury) and type II (formerly known as Causalgia, with proven nerve injury). A distinction can be made between initially “cold” and “warm” CRPS [6]. CRPS presents itself with a disproportional pain, accompanied by changes in the following categories: sensory (allodynia, hyperalgesia), vasomotor (temperature asymmetry, skin color changes or asymmetry), sudomotor (edema, sweating changes or asymmetry), and motor/trophic (decreased range of motion, motor dysfunction, trophic changes) [7]. CRPS almost exclusively occurs on the distal extremities after major or even minor injury, and spreading has been rarely reported [8]. The disease’s course is dynamic, with potentially changing clinical appearance over time. Usually, the initial features include disproportionate pain and swelling distal to the site of injury within 8 weeks of the initial injury [9, 10]. Often these features are accompanied by hyperthermia and redness, which is most likely due to autonomic nervous system dysfunction. Over time, atrophy and dystrophy may occur, secondary to the inflammatory changes. The onset of the first CRPS symptoms usually begins within days to weeks. Disproportionate pain commonly occurs together with persistent swelling: In one investigation, pain or autonomic symptoms were described by 75% of patients within one day [11], in another study 67.3% of patients demonstrated autonomic phenomena in the first week and 94.2% within the first month [12•].

Risk factors are female sex, immobilization, nerve damage, (open) fractures, asthma, migraine, osteoporosis, rheumatoid arthritis, ACE inhibitors, and a tight cast after fracture [12•, 13–19]. Further, there is a proneness to developing CRPS later in life if it has already occurred in the past [20]. However, a severe injury does not automatically lead to a more severe CRPS, as these are rather expected in cold CRPS and in injuries of the upper extremity [21].

Emotional distress due pain and/or interference with activities of daily living and social participation are often present [22••]. As a result, the high association with psychiatric comorbidities has led to the hypothesis of a “Sudeck personality,” but this has ultimately never been proven [23]. On the contrary, studies suggest that maladaptive personality traits are much more likely to develop as a consequence rather than a cause of the pain [24]. For example, a recently published study found that negative affect (catastrophizing, anxiety, and depression) or sleep disturbances did not predict long-term CRPS — but more widespread pain symptoms and pain intensity did [25••]. Similarly, high posttraumatic/postoperative pain scores have long been associated with the development of CRPS: in one investigation, it was demonstrated that disproportionate pain of at least 5 points on a numerical rating scale (0 = no pain at all, 10 = worst possible pain) within the first week after trauma led to an increased risk of CRPS [26]. Conversely, a recent study highlighted that strong pain was not a predictor of CRPS duration [27••]. While there is evidence for the predictive value of quantitative sensory testing to predict acute pain for different surgical interventions [28–30], no respective data exist for the development of CRPS. Thus, the exact role of pain intensity is still being debated.

## Pathophysiology

In the beginning, a drop in norepinephrine levels in the circulation of the affected limb can cause the typical presentation with hyperthermia and redness [31]. Afterwards, a peripheral catecholamine receptor sensitization may lead to late-phase peripheral cyanosis [32]. The tissue injury provokes a local inflammatory response with an increase in proinflammatory cytokines. Greater concentrations of IL-1, IL-6, TNF-α, substance P, and bradykinin are seen, which increase nociceptor sensitivity and promote neurogenic inflammation [33, 34]. Further, increased signal transduction occurs in the dorsal horn because of elevated peripheral nerve activation. Nociceptive and adrenergic neuronal coupling may lead to sympathetically maintained pain, while cortical remodeling occurs at the level of the CNS [35]. Frequently, limited representation of the afflicted limb on the somatosensory cortex accompanies these phenomena [36] and can lead to restricted movement, disturbed own body perception, and impaired tactile presentation. Clinically, contralateral sensitization is frequent [37]. Furthermore, animal models have demonstrated the important role of an autoinflammatory (see above) and immune component [34]. For example, a group of researchers recently induced CRPS-like signs and mechanical hyperalgesia in mice by passive transfer of purified serum immunoglobulin G (IgG) from CRPS patients. However, it is still unclear why only one limb is often affected in patients with autoimmune-related pain. These open questions will have to be answered in further studies.

## Diagnosis

**Fig. 1:**
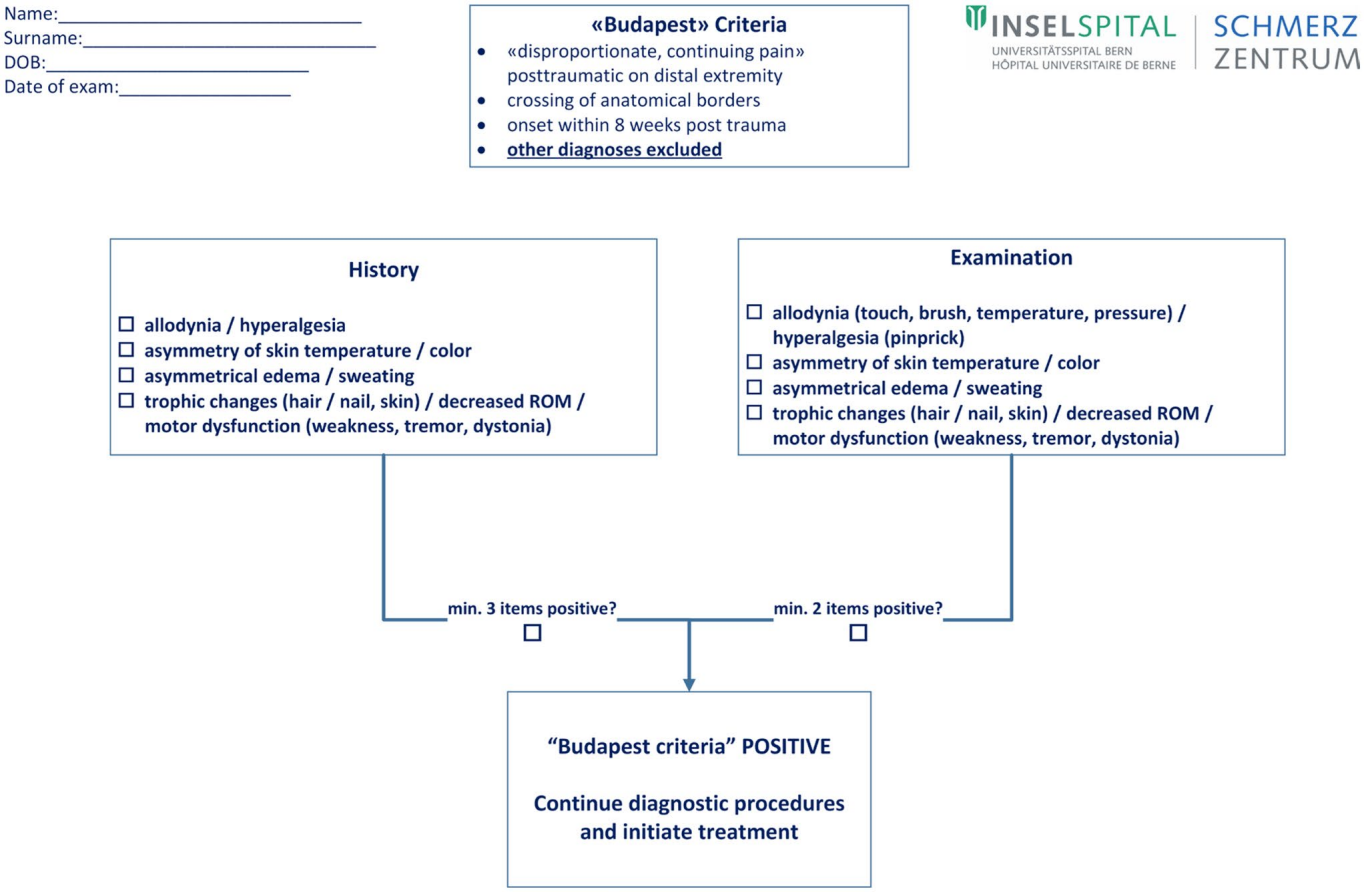
Diagnostic sheet used at Department of Anaesthesiology and Pain Medicine, Inselspital, Bern University Hospital, Switzerland, based on the New IASP diagnostic criteria for CRPS, “Budapest criteria” [7]

It is crucial to remember that the CRPS characteristics can occur in a variety of combinations and only continuous, disproportionate pain is mandatory. Further, it should be noted that none of the CRPS characteristics are pathognomonic. Therefore, clinicians must first rule out other differential diagnoses (Table 1), before they can use the New IASP Diagnostic Criteria for CRPS (“Budapest Criteria”) [7]. This is done by combining symptoms (i.e., patient-reported phenomena) and signs (clinically observed/examined features) in 4 different categories: sensory, vasomotor, sudomotor/edema and motor/trophic. At least one symptom in three of these categories needs to be reported and at least one sign in at least two categories observed. A diagnostic sheet based on the “Budapest criteria” is attached in Fig. 1. Recently, the IASP CRPS Special Interest Group was convened in 2019 to clarify some criteria to avoid misunderstandings in clinical practice and research [22••]. For example, hyperalgesia should be understood as an amplification of an inherently (low) painful stimulus, whereas allodynia is the painful sensation of a non-painful stimulus. Regarding symptoms, patients must be asked specific questions addressing each category, and it is not enough to just ask open-ended questions. Asymmetries should be recorded as side-to-side changes evident to the patient. In the clinical examination, hyperalgesia should be tested as a pinprick in the center of the affected area; assessment of allodynia includes superficial touch, cold/warm temperature, pressure (enough to turn the examiner’s fingernail bed color white), and vibration. Cases lasting for more than 12 to 18 months can be considered having “persistent” CRPS [22••]. The term “chronic” should be avoided to prevent misunderstanding with “chronic pain” (i.e., pain lasting longer than 3 months). In the new International Classification of Diseases, 11th Revision (ICD-11), which went into effect January 01, 2022, CRPS is coded with primary pain disorder as parent diagnosis (MG30.04).

**Table 1:** Potential differential diagnoses of CRPS

**Neuropathies**	Injuries or compression of roots, plexus, or singular nerves Peripheral neuropathy
**Vascular disorders**	Atherosclerosis Chronic venous insufficiency
**Inflammations/infections**	Low-grade infections Arthritis Tendinitis
**Rheumatologic disorders**	Myofascial pain Tendinitopathies
**Osseous causes**	Osteomyelitis Pseudarthrosis Failure of osteosynthetic material Necrosis Osseus/other fragments
**Psychiatric causes**	Self-mutilation Bodily distress disorder

“Technical” examinations are primarily performed to rule out potential differential diagnoses and to confirm CRPS. Unfortunately, there is no gold standard test to rule out other pathologies, and a multitude of efforts have resulted in mixed results for many apparative procedures. For instance, there has been a negative study for Quantitative sudomoter axon reflex test (QSART) [38], and small sample sizes limit the informative value of procedures like laser Doppler imaging [39] or the thermoregulatory sweat test (TST) [40]. Many of these methods have therefore to be considered experimental; further, they are often expensive and only available in super-specialized centers. In contrast, plain radiographs can be used to diagnose missed fractures or facilitate the diagnosis of CRPS as soon as bony changes (usually within 3–12 months) develop [41]. There is, however, the risk of confounding with disuse atrophy. Further, a 3-phase bone scintigraphy with technetium-99 m diphosphonate shows a high specificity of 83–100% (few false positive cases), but its sensitivity only lies at 31–50% (many false negative cases) [42], making it impossible to rule out CRPS if the result is negative. Further examinations include skin temperature side differences of > 2 °C [43], which can be detected by clinical examination and orienting thermography and confirmed by subsequent transcutaneous follow-up measurements. In cases with CRPS II, it is necessary to prove a nerve compression or injury, for instance, using electroneurography, magnetic resonance imaging, or ultrasound. As noted above, the displayed features must extend beyond the border of any nerve distribution area.

## Treatment

The most important treatment of CRPS is early recognition and initiation of comprehensive care. Therapy itself is similarly complex to the diagnostic process and must be adapted to the patient. Precisely because treatment can be extensive and sometimes complicated, patients need to be educated and informed about the condition and the possible course of the disease. Cases that do not show a rapid improvement within 2 months after the onset of symptoms must be referred to specialized centers [44]. Although there are individual randomized controlled trials (RCTs) for certain treatments, the literature is often inconsistent and rather weak for many therapies [45]. The following recommendations are based on the 2018 S1-guideline of the German Society of Neurology [46] and its update of 2019 [47], as well as on the NICE guidelines for the treatment of CRPS [48]. The broad therapy goals are analgesia, which coincides with the most important aim of patients [49], and restitution of function. Further, psychological support and the stabilization of the socioeconomic situation are crucial pillars in the care of persistent CRPS cases. Therapies can be broadly divided into four categories: drug therapy, invasive, body-oriented, and psychological/behavioral. A summary and suggested “checklist” for a reasonable therapy plan is outlined in Table 2.

**Table 2:** Proposed therapy overview

**Treatable triggers excluded?**	For example, nerve compression in CRPS II Differential diagnoses excluded? X-ray? 99mTc scintigraphy? Repeated temperature measurements?
**Analgesic therapy: stepwise trial of different drugs**	Tricyclic antidepressants (amitriptyline, surmontil) Anti-epileptics (pregabaline, gabapentine)
**Evaluation of therapies with time frame**	High-dose cortisone (up to 6 months) Bisphosphonates (in acute CRPS, preferably 3–4 months)
**Body-centered therapy**	Ergotherapy (mapping, allodynography) Somatosensory rehabilitation (graded motor imagery) Physiotherapy with behavioral components; EXP
**Invasive therapy**	Sympathetic block trial, if positive: 4–10 blocks Trial of i.v.-ketamine SCS/DRG in therapy resistant cases, assessment within 2 years
**Psychotherapy**	Assessment of psychiatric comorbidities and treatment Patient education, training of body awareness Cognitive behavioral therapy
**Socioeconomic assessment**	Return to work realistic? If yes, to what degree? Insurance claims? Re-education needed? Disability Insurance involved? Counseling by social service

### Drug Therapy

There is no data supporting the use of non-steroidal anti-inflammatory drugs for CRPS specifically; however, these drugs are often given to interrupt the initial inflammatory phase. Drug treatment follows that for neuropathic pain with a few exceptions. In general, medications are classified as off-label use, which should be communicated to patients. For the classical antineuropathic drugs, there is one RCT each for amitriptyline [50] and gabapentine [51], with dosing similar to that for neuropathies. Gabapentinoids and tricyclic antidepressants are commonly used. Furthermore, oral steroids can be used, which, as strong anti-inflammatory agents, are intended to interrupt the inflammatory processes, especially at the beginning of the disease. In two RCTs [52, 53], relatively low doses were used, but in clinical use, higher doses of 100 mg prednisolone daily, phased out over two weeks, were often recommended [46]. Concomitant administration of steroids with NSAIDs must be avoided due to the potential gastrointestinal side effects. Rarely, flushing, insomnia, weight gain, or euphoria can occur during steroid pulse therapy. This treatment is generally recommended within the first 6 months. A similar time limit exists for bisphosphonates, for which five RCTs have been conducted to date [54–58]. These substances inhibit osteoclast activity and thus bone resorption, but also have an anti-inflammatory effect and modulate spinal microglia. Again, use within the first 6 months is considered effective, and — hypothetically — patients with proven accumulation of technetium-99 m in 3-phase bone scintigraphy could benefit most. However, the administration of bisphosphonates is associated with potential side effects such as nausea, gastrointestinal reflux and indigestion, flu-like symptoms, flushing, and muscle pain. The most feared adverse event is the osteonecrosis of the jaw, which is why dental examination is recommended before treatment. Accordingly, this therapy belongs in the hands of specialists.

As in other inflammatory processes, free oxygen radicals play an important role in CRPS. They are supposed to be treated by the administration of vitamin C. One RCT proved the prophylactic benefit of the drug [59], and this preventive effect has been confirmed in several systematic reviews and meta-analyses [60, 61•, 62]. Due to the favorable side effect profile and the low price, the administration of vitamin C (500 mg daily for 50 days) is easy to perform and can be generally recommended in patients at risk of developing CRPS. Topical dimethyl sulfoxide (DMSO) is said to have a similar mechanism of action, scavenging radicals. However, a first RCT on this was insufficiently blinded [63], a second study had N-acetylcysteine as a control group [64], which allows either the conclusion that both substances are equally efficient or equally inefficient for the therapy of CRPS [46]. Usually, DMSO is prescribed as 50% cream 5 times daily for 3 months, and side effects are rarely reported.

### Invasive Therapies

Because of the possible side effects and the necessary monitoring during the application, these therapy forms belong in specialized centers. Ketamine is a substance of interest, best known for its NMDA antagonism but also for modulation of cholinergic neurotransmission and enhancement of descending modulatory pathways. When used, ketamine produces very rapid pain relief, especially in neuropathic pain, and is also thought to counteract central pain sensitization, although this effect has not been well studied. For ketamine, there are two RCTs (one with a continuous infusion over 24 h for 4 days and one with an infusion for 4 h over 10 days), which resulted in a pain reduction for up to 12 weeks [65, 66]. In vivo models show a positive effect of ketamine in the persistent, but not the acute phase of CRPS [67]. Sympathetic blocks suppress the sympathetic nervous system and can lead to pronounced pain relief. However, the most recent Cochrane Review was unable to make a recommendation on this treatment due to poor data [68]. There are three RCTs, two of which studied ganglion stellate blocks [69, 70] and one investigated thoracic sympathetic blocks [71]. A recent retrospective analysis suggests performing a test block in the absence of contraindications and performing four to ten blocks as a series if the result is positive (≥ 50% pain relief) [72•]. Furthermore, spinal cord stimulation (SCS) may be considered in patients who have not benefited adequately from other therapies. The hyperexcitability of wide dynamic range neurons is reduced by means of an electrode (or electric field). New stimulation protocols could provide additional benefit, as was recently highlighted in a case study where an SCS with 10-kHz high frequency (HF10) stimulation led to 75% pain relief as well as a reduction in erythema, hyperthermia, and edema [73]. Testing should be performed prior to implantation and evaluation should occur within the first two years of CRPS diagnosis. There are two major RCTs for SCS [74, 75], and interestingly, different protocols were preferred by patients. Therefore, the adaptation of the stimulation form should be done individually. In addition, an RCT for dorsal root ganglion stimulation was published a few years ago [76], which provided significantly better results in CRPS of the lower extremity.

### Body-Oriented Treatment

Physiotherapy should not only prevent pathological movement patterns and “learned non-use” of the affected limb, but also train behavioral aspects. Including active physiotherapy is essential, as passive measures alone are unsuitable to treat CRPS. Immobilization and restriction should be avoided if possible [15]. A special physiotherapy form is the “Exposure In Vivo” (EXP), where fear-inducing activities are first identified and then addressed by repetitive exposure in a protected, therapeutic setting with the aim to reduce the threat value. There is one RCT for a treatment of 17 weeks, which showed less pain and impairment and more function of the affected limb [77]. It is important to note that these exercises should never be performed without consent and understanding of the patient. To address altered central processing, mirror therapy and graded motor imagery (GMI) can normalize the interaction of sensor and motor functions and reduce anxiety in using the affected extremity. For GMI, two RCTs indicated positive results, while the latest multicenter RCT yielded negative results in terms of pain relief [78]. Occupational therapy can be trialed to reduce painful movement patterns, normalize sensibility, and desensitize allodynia-affected areas; two RCTs attest a positive effect on impairment [79, 80].

### Psychological/Behavioral Therapy

Psychotherapy aims at treating psychiatric comorbidities, reduce anxiety, and promote relaxation and imagination techniques. Further goals are to improve body perception and self-awareness. Since CRPS is “complex” in nature, patients (and sometimes healthcare providers) can feel overwhelmed with diagnosis and treatment. It is important to emphasize that the illness is not psychogenic, but that educational aspects and self-empowerment must be addressed [81]. It is crucial to enable patients to reframe their own participation in active rehabilitation and reverse dysfunctional behavior or catastrophizing. For CRPS, there are few specific studies of cognitive behavioral therapy, but one study of a small cohort of six patients was able to demonstrate normalization of somatosensory cortices, pain reduction, and improvement in tactile discrimination [82].

## Importance of Early Detection of CRPS

Because of the early onset of CRPS symptoms and signs in the vast majority of cases, one would expect a rapid diagnosis. In clinical reality, however, much longer latencies between injury and diagnosis are observed, depending on the study. For example, a prospective analysis found that after removal of the cast (i.e., 6 weeks after fracture), it took an additional 21.7 ± 23.7 days (corresponding to an overall mean time of nine weeks after tissue damage) for the diagnosis of CRPS in conservatively treated radius fractures [83]. Another Scandinavian study of 52 patients found a mean delay of 33.5 months (2.8 years) between injury and diagnosis [12•]. Symptoms and signs preceding the diagnosis were also rarely documented: severe pain was recorded in only 10 of 34 cases and autonomic abnormalities in 51–60% of patients during the first four months after tissue damage. Overall, these findings indicate a lack of awareness of the risk of CRPS after tissue injury of the extremities.

Complicating matters further, many practitioners struggle with diagnosis and treatment. For example, a 2016 international survey showed that 50% of CRPS specialists surveyed reported at least “some” difficulty in recognizing symptoms and signs of CRPS [84]. Extrapolated to the general population of primary care providers, making an informed diagnosis and establishing a structured treatment plan may be even more difficult. It is certainly a challenge to distinguish a delayed healing processes from a beginning CRPS, especially in the 5–10% of patients who do not show apparent tissue damage [11, 44].

There are several strategies to mitigate negative patient outcomes and prevent unfavorable socioeconomic impacts:

1. Prevention of CRPS
  Early recognition of the first signs of CRPS can prevent a fully developed CRPS and reduce the severity and duration. In a cohort of patients with conservatively treated distal radius fractures, there were significantly fewer cases of CRPS after staff training on symptoms/signs of a beginning CRPS and cast management were implemented [15]. As mentioned before, disproportionate pain [26], persistent swelling, or a pressing cast [15] may be indications of beginning CRPS that justify early, pro-active therapy. There is a lack of knowledge about evidence for early prognostic factors associated with the progression of CRPS [85•].
2. Rapid Escalation of Therapy in Refractory Cases
  The greatest therapeutic success can be expected within the first months of the disease [44, 86], yet only about 0–5% of patients are symptom-free after 1 year [10, 87]. Further, a cluster analysis showed that about one in seven patients will not improve 1–2 years after trauma [21], and other studies have found that treatment becomes increasingly difficult the longer CRPS persists [11, 88]. This has also been shown during the validation of the newly developed CRPS severity score, where patients with an already persistent CRPS showed no statistically significant improvement after 3 months, despite extensive, multimodal treatment [89]. Therefore, in the absence of rapid relief, prompt expansion of therapy and referral to specialized or “superspecialized” centers (i.e., with expertise in invasive neuromodulation) should occur quickly [44].
3. Prevention of Somatic and Psychological Comorbidities
  The likelihood of somatic or psychological comorbidities increases with the duration of untreated CRPS [88]. Somatic comorbidities include dystonia and infections, which sometimes provide the indication for invasive treatments [90]. The psychological symptoms that often accompany CRPS include catastrophizing, depression, anxiety, stress, and body perception disturbances. As in most chronic and complex conditions, patient education is of high importance. The burden of somatic and psychological symptoms often leads to ongoing problems, as some patients may not be able to work or need assistance for their activities of daily living. For example, according to a retrospective study, 9% required help with their personal hygiene [91]. All of these areas must be addressed in comprehensive care.
4. Prevention of Reoccurring CRPS
  Reoccurence in a patient with a history of CRPS is an understudied topic, but the risk seems to be substantial. In one investigation, 13% (6 of 47 patients) developed another CRPS postoperatively, although only one case was serious and persistent [92]. Further, long surgery duration of over 180 min is associated with the resurgence of the disease (odds ratio 5.7) [93]. As mentioned above, solid data for prevention of CRPS is only available for vitamin C. Expert opinion suggests the implementation of a multimodal regimen [94] with the use of at least two non-opioids [95] and possibly including a ketamine perfusion where the NMDA antagonism could help block neuropathic pain. Pathophysiologically, regional anesthesia could be useful to interrupt nociceptive triggers and promote sympathicolysis. Surgery should be performed by experienced surgeons and anesthetists, with minimally invasive procedures if possible and reduced tourniquet time.
5. Health and Economic Costs
  According to one prospective study, no significant improvements in the disability or work status were recorded after 6 months [87] and in another investigation 31% of patients with persistent CRPS were unable to work [21]. These high morbidity rates result in a high impact with one publication placing the average insurance costs within 5 years at ~ $87.000 and treatment costs at ~ $23.000 [96]. Early socioeconomic assessment is therefore critical to (1) determine whether problems in maintaining income are already apparent and (2) to initiate counseling and registration with the relevant social insurances.

## Conclusion

In the last decade, significant advances have been made to streamline the diagnosis of CRPS and coordinating research. The “Budapest criteria” are now a standardized assessment and are being applied in clinical and research settings worldwide. Moreover, the Valencia Consensus has provided clarity in their application. However, the suspected CRPS subtypes and the role of the autoimmune system need further research to finally provide tailored therapy for subgroups of patients. In the meantime, the greatest challenge in the clinical context is to create an awareness of the risk of CRPS, to detect the disease early using standardized diagnostic processes, and to rapidly expand therapy in refractory cases. In addition, comorbidities and socioeconomic factors must be addressed early to prevent negative consequences for patients.

## Data Availability

Not applicable.

## Abbreviations

CRPS: Complex regional pain syndrome
CSS: CRPS severity score
EXP: Exposure in vivo therapy
GMI: Graded motor imagery
HF10: 10-KHz high frequency
IASP: International Association for the Study of Pain
ICD-11: International Classification of Diseases, 11th Revision
IgG: Immunoglobulin G
QSART: Quantitative sudomoter axon reflex test
RCT: Randomized controlled trial
SCS: Spinal cord stimulation
TST: Thermoregulatory sweat test
